# *Lactobacillus helveticus* mitigates diarrhea and inflammation induced by enterotoxigenic *E. coli* through rebalance of gut microbiota

**DOI:** 10.1016/j.crfs.2025.101147

**Published:** 2025-07-17

**Authors:** Zhen Zhang, Jianmin Lv, Xin Wang, Ling Chun, Qiannan Yang, Huarui Zhao, Siming Xue, Ziyi Zhang, Xiaobo Liu, Shiwei Wang, Yanmei Sun

**Affiliations:** a Key Laboratory of Resource Biology and Biotechnology in Western China, Ministry of Education, Provincial Key Laboratory of Biotechnology, College of Life Sciences, Northwest University, 229 Taibai North Road, Xi'an, Shaanxi 710069, China; b Rehabilitation Science Institute, Shaanxi Provincial Rehabilitation Hospital, Xi'an, Shaanxi, 710065, China; c Key Laboratory of Metabolic Engineering and Biosynthesis Technology, Ministry of Industry and Information Technology, Nanjing University of Science and Technology, Nanjing, Jiangsu, 210094, China

**Keywords:** *Lactobacillus helveticus*, Enterotoxigenic *Escherichia coli*, Diarrhea, Gut microbiota, Short-chain fatty acids

## Abstract

The probiotic potential and mechanisms of *Lactobacillus helveticus* against *Escherichia coli* (ETEC) induced diarrhea remain poorly understood, despite its robust physiological traits and long history of safe use. Here, we compared the anti-diarrheal efficacy of four *L. helveticus* strains (H1-H4) isolated from different geographical origins to unravel the mechanisms of action in ETEC-infected mice. Surprisingly, feeding with *L. helveticus* strains significantly reduced pro-inflammatory cytokines tumor necrosis factor-α (TNF-α), interferon-γ (IFN-γ) and interleukin-6 (IL-6), alleviated jejunal damage, and restored the colon length. Among them, *L. helveticus* H3 exhibited the best protective effects through upregulation of aquaporin 3 (AQP3) and interleukin-10 (IL-10) and downregulation of heat-stable enterotoxin (ST) and toll-like receptor 4 (TLR4). Notably, ETEC infection disrupted intestinal homeostasis of microbiota by enriching *Akkermansia* and *Escherichia-Shigella* and suppressing *Lactobacillus*, *Odoribacter*, *Alistipes*, *Ligilactobacillus*, and *Bifidobacterium*; whereas the supplementation with *L. helveticus* could effectively restore the balance of gut microbiota. Moreover, the strain H3 significantly enhanced the production of several short-chain fatty acids (SCFAs), including butyric, propionic, isobutyric, valeric, isovaleric, and caproic acids, compared to the others. Comparative genome analysis revealed that the strain H3 had more genes that likely contributed to SCFA production, toxin detoxification, and colonization capacity, which support its better efficacy in alleviating ETEC-induced diarrhea. Collectively, our study suggests that *L. helveticus* alleviates ETEC-induced diarrhea by rebalancing gut microbiota and is a promising probiotic for treating infectious diarrhea.

## Highlights

• *Lactobacillus helveticus* strains from artisanal yogurts alleviate infection-related diarrhea.
• Strain H3 shows superior efficacy by restoring colon length and mucosal integrity.
• H3 enhances short-chain fatty acid levels and reduces inflammatory cytokines.
• Genomic analysis links H3's effects to unique genes in metabolism and colonization.
• Infection-driven overgrowth of *Akkermansia* suggests context-dependent pathogenicity.

## Graphical abstract

**Figure:**
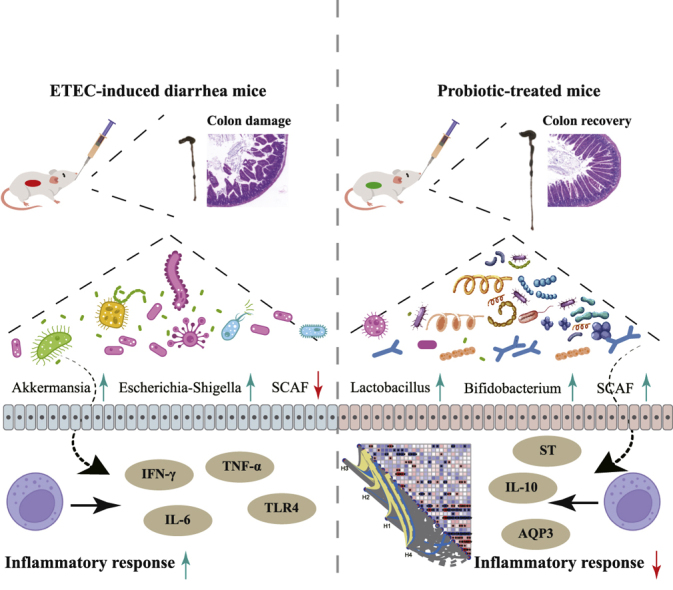

## 1 Introduction

Enterotoxigenic *Escherichia coli* (ETEC) is a major pathogen responsible for severe diarrheal diseases, posing significant global health and economic burdens. In humans, ETEC is a leading cause of travelers' diarrhea and infantile diarrhea in low-resource regions, contributing to an estimated 380,000 deaths annually in children under five years of age (Fleckenstein et al., 2010; Reiner et al., 2018). In livestock, ETEC infections in intensive farming systems lead to high morbidity and mortality in neonatal calves and weaned piglets, adversely affecting animal welfare and productivity, and driving the overuse of antibiotics in agriculture (Fu et al., 2024; von Mentzer and Svennerholm, 2024). The virulence of ETEC is primarily attributed to host-specific colonization factors, such as human CFA/I and porcine K88/K99 fimbriae, as well as enterotoxins (LT/ST) that disrupt intestinal ion transport, induce inflammation, and cause potentially fatal secretory diarrhea (Smith et al., 2022; Wang et al., 2019).

Antibiotics, including fluoroquinolones, macrolides, and sulfonamides, have long been the standard treatment for ETEC-induced diarrhea (DuPont and Mattila, 2003). While they are effective in reducing bacterial load and alleviating symptoms, their widespread use has contributed to the emergence of antimicrobial resistance, posing a public health threat (Miller and Arias, 2024). Additionally, antibiotics can cause adverse effects such as gastrointestinal discomfort, dizziness, and disruption of the gut microbiota. Prolonged use may lead to immune dysfunction, liver damage, and an increased risk of secondary infections (Virk et al., 2013). In neonates and infants, whose immune systems and gut microbiota are still developing, antibiotics increase susceptibility to dysbiosis and long-term microbiota imbalances. In livestock, antibiotic abuse in intensive farming systems fosters resistant strains that can be transmitted to humans through the food chain, further complicating infection management (Vangay et al., 2015; Woolhouse et al., 2015).

Probiotics, especially species within *Lactobacillus* and *Bifidobacterium*, have emerged as promising alternatives to antibiotics for mitigating ETEC-induced pathology. Their benefits include enhancing mucosal immunity, reinforcing intestinal barrier function, modulating gut microbiota, and inhibiting pathogen colonization and enterotoxin production (De Keersmaecker et al., 2006; Yang et al., 2021; Yue et al., 2020). Numerous *Lactobacillus* strains have demonstrated anti-inflammatory and gut-protective properties. For instance, *L. plantarum* ZLP001 upregulated tight junction proteins and enriched SCFA-producing microbes in piglets (Wang et al., 2018), while *L. gasseri* and *L. rhamnosus* GG alleviated ETEC-induced diarrhea through immune modulation and microbiome restructuring (Roselli et al., 2006; Chen et al., 2023).

Despite these advances, the probiotic potential of *L. helveticus* remains largely underexplored in the context of infectious diarrhea. Notably, *L. helveticus* possesses several advantageous features, including robust acid and bile tolerance, a highly active proteolytic system that generates bioactive peptides with immunomodulatory and mucosal-protective functions, and a long history of safe use in dairy fermentation. These attributes suggest its untapped potential as a targeted therapeutic against enteric infections. However, systematic evaluation of its strain-specific efficacy and mechanisms in ETEC models is lacking.

In this study, we addressed this critical knowledge gap by investigating four *L. helveticus* strains (H1–H4) isolated from traditional fermented foods in underrepresented ethnic regions of China. We demonstrated that these strains, particularly H3, exhibit distinct protective effects against ETEC-induced diarrhea. Beyond classical endpoints such as inflammation and SCFA levels, our study introduced colon length as a novel morphological marker of infection severity. Moreover, we identify a striking, context-dependent enrichment of *A*. *muciniphila*, a typically beneficial microbe, which may play a deleterious role under dysbiotic conditions. This observation underscores the functional plasticity of *A. muciniphila* and highlights the importance of evaluating probiotic behavior within disease-specific contexts, as host–microbiota interactions can shift from mutualistic to pathogenic depending on physiological status. These findings offer new insights into probiotic strain selection, microbiota–pathogen dynamics, and host response, and indicate *L. helveticus* as a promising candidate for next-generation probiotic therapies.

## 2 Materials and methods

### 2.1 Cultivation and preparation of bacterial strains

Four *L. helveticus* strains were isolated from traditionally fermented yogurts produced by ethnic minority communities in various regions of China. They were subsequently deposited in the Culture Collection of Microorganisms at Northwest University, Xi'an, China (Table 1). Each strain was subcultured three times in de Man, Rogosa, and Sharpe (MRS) broth at 37 °C for 16 h. *L. rhamnosus* GG, which is a well-established probiotic known to alleviate diarrhea (Tang et al., 2019; Vanderhoof and Young, 1998), was used as the positive control. The four *L. helveticus* strains along with *L. rhamnosus* GG, were cultured in MRS medium at 37 °C at 200 rpm for 24 h. After incubation, bacterial strains were harvested by centrifugation at 6000×*g* for 20 min at 4 °C. The bacterial pellets were washed twice with ice-cold phosphate-buffered saline (PBS) and resuspended in a sterile protective medium containing 13 % skim milk, 2 % trehalose, and 2 % sucrose (1:2, m/v). The suspensions were then freeze-dried, sealed in airtight containers, and stored at 4 °C. The final concentration of viable bacterial cells in each suspension was adjusted to 5 × 10^9^ CFU.

**Table 1:** Strains used in this study.

Strain	Origin	Region	Genome size
*L. helveticus* H1	Handmade Dairy	Rangtang, Sichuan Province	1844 kb
*L. helveticus* H2	Handmade Dairy	Aba, Sichuan Province	1836 kb
*L. helveticus* H3	Handmade Dairy	Aba, Sichuan Province	1990 kb
*L. helveticus* H4	Handmade Dairy	Nagqu, Xizang Province	1783 kb

*Escherichia coli* ETEC O78:K80 (CICC 10421), obtained from the China Center of Industrial Culture Collection (CICC, Beijing, China), was selected due to its well-documented virulence and widespread use in murine models of diarrheal disease. This strain expresses heat-stable (ST) and/or heat-labile (LT) enterotoxins, closely mimicking the pathogenic features of clinical human isolates, and has been shown to induce consistent diarrheal symptoms in mice (Read et al., 2014). For infection, the strain was cultured in Luria–Bertani (LB) broth at 37 °C with shaking at 180 rpm for 18 h. Cultures were centrifuged at 4000×*g* for 10 min at 4 °C, and the resulting bacterial pellets were washed twice with sterile phosphate-buffered saline (PBS). The final suspension was adjusted to 1.5 × 10^11^ CFU/mL for animal administration.

### 2.2 Animals and experimental design

Four-week-old female specific-pathogen-free (SPF) BALB/c mice were obtained from Chengdu Dossy Experimental Animal Co., Ltd. (Sichuan, China). All animal procedures adhered to the guidelines set by the Laboratory Animal Management Committee of the Ministry of Health of the People's Republic of China. The study protocol was approved under the approval number NWU-AWC-20241204Z. Mice were maintained in a SPF facility at 25 ± 2 °C and 55 ± 5 % relative humidity, with a 12-h light/dark cycle. They were provided with ad libitum access to sterile food and water throughout the experiment.

**Fig. 1:**
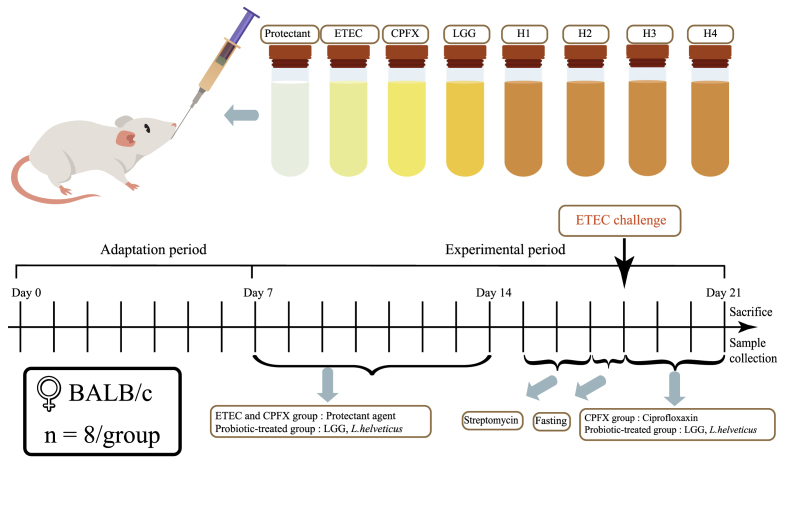
Design of animal experiments. Sixty-four female BALB/c mice were acclimatized for seven days and randomized (n = 8) into Control, ETEC, CPFX, LGG, and *L. helveticus* H1–H4 groups. Days 1–7: Control, ETEC, CPFX received vehicle gavage; LGG and H1–H4 received 200 μL probiotic. Days 8–10: 5 g/L streptomycin in drinking water. Days 11–13: after an 18 h fast, all but Control were challenged twice daily with 200 μL ETEC. Days 11–14: CPFX received ciprofloxacin; LGG and H1–H4 continued probiotics; Control and ETEC received vehicle. On day 14, intestinal tissues and cecal contents were collected for histology, cytokine, and microbiome analyses.

After a one-week acclimatization, 64 female BALB/c mice were randomly assigned to eight groups: control, ETEC, ciprofloxacin (CPFX), *Lactobacillus rhamnosus* GG (LGG), and four *L. helveticus* treatment groups (H1–H4) (Fig. 1). From days 1–7, mice in the control, ETEC, and CPFX groups received daily oral gavage of 200 μL protectant solution as vehicle control. Probiotic groups were gavaged daily with 200 μL freshly prepared suspensions of *L. helveticus* strains (5 × 10^9^ CFU/mL), while the LGG group received an equal volume and concentration of LGG (ATCC 53103). All gavage procedures were performed at the same time each day using sterile flexible feeding needles. Mice were monitored daily for health and behavior to ensure physiological stability before infection.

To facilitate consistent colonization by ETEC, all mice were administered streptomycin (5 g/L) in drinking water for three consecutive days (days 8–10) to transiently deplete gut microbiota. This antibiotic pretreatment disrupts commensal bacterial communities, reducing colonization resistance and enabling reproducible infection (Yang et al., 2021; Zhang et al., 2022). To model acute enteric infection and short-course therapeutic intervention, the dosing regimen was adapted from established murine diarrhea protocols (Han et al., 2021; Vidal et al., 2010). From days 11–14, all groups except the blank control received *E. coli* ETEC O78:K80 suspension (1.5 × 10^11^ CFU/mL) by oral gavage twice daily. *L. helveticus* and LGG groups received an additional daily gavage containing the probiotic suspension, while the antibiotic group was administered ciprofloxacin (5 g/L) once daily. Blank controls were given sterile saline three times daily. This design ensured consistent gavage frequency across groups and minimized procedural variability while capturing the dynamics of acute infection and intervention.

### 2.3 Measurement of body weight and fecal moisture content

Throughout the experiment, body weight and food intake were recorded daily for each mouse. Fecal samples were collected individually before euthanasia, and fecal moisture content was quantified by freeze-drying, as described previously (Julio-Pieper et al., 2010). Mice were anesthetized with 1 % isoflurane and euthanized by cervical dislocation. The jejunum was then harvested, fixed in 4 % paraformaldehyde, and processed for dehydration and embedding according to standard protocols (Julio-Pieper et al., 2010). Ultrathin sections (5 μm) of the jejunum were stained with hematoxylin and eosin (H&E) and subsequently scanned using a Panoramic MIDI scanner (3DHISTECH Ltd., Budapest, Hungary). Villi length and crypt depth were subsequently measured using the CaseViewer software.

### 2.4 Assessment of immune factors

Blood samples were collected from mice and centrifuged at 4000×*g* for 10 min to separate the serum, which was then aliquoted and stored at −80 °C for subsequent analyses. cytokines TNF-α, interferon-γ (IFN-γ), and IL-6 in the serum were quantified using commercial ELISA kits (FANKEW; Shanghai Kexing Trading Co., Ltd.) according to the manufacturer's instructions. Levels of the cytokine IL-10 and protein aquaporin-3 (AQP3) in colon tissue were determined homogenization using a high-throughput tissue disruptor. In addition, the serum levels of the heat-stable enterotoxin (ST) and the receptor Toll-like receptor 4 (TLR4) were also measured using FANKEW ELISA kits (Shanghai Kexing Trading Co., Ltd.), strictly adhering to the provided protocol.

### 2.5 Microbial composition analysis of gut microbiota

Fecal pellets were freshly excreted and non-invasively collected from each mouse immediately before euthanasia to reflect global microbial shifts induced by ETEC infection. Samples were handled aseptically, snap-frozen in liquid nitrogen within minutes of collection, and stored at −80 °C until microbial DNA extraction, in accordance with previously validated protocols (Yang et al., 2021; Chen et al., 2023). Total microbial genomic DNA was extracted from the fecal samples using the FastDNA SPIN Kit (MP Biomedicals, LLC, Irvine, CA, USA) following the manufacturer's instructions. The V3–V4 hypervariable regions of the bacterial 16S rRNA genes were amplified using universal primers 338F (5′-ACTCCTACGGGAGGCAGCA-3′) and 806R (5′-GGACTACNNGGGTATCTAAT-3′), with primer sequences and PCR conditions based on previously established methods. PCR products were separated by agarose gel electrophoresis, purified using the QIAquick Gel Extraction Kit (Qiagen GmbH, Hilden, Germany), and quantified using standard methods (Sun et al., 2019). The purified PCR products underwent paired-end sequencing (2 × 300 bp) on the Illumina MiSeq platform (Illumina Inc., San Diego, CA, USA), following validated sequencing protocols. Raw sequencing data were processed with the QIIME 2 pipeline for quality control and feature table generation. Alpha diversity indices (Chao1 richness and Shannon diversity) and Non-metric multidimensional scaling (NMDS) were assessed using the MicrobiomeAnalyst online platform (https://www.microbiomeanalyst.ca). Furthermore, differences in microbial community composition between groups were analyzed using LEfSe via the online Galaxy platform (http://huttenhower.sph.harvard.edu/galaxy/).

### 2.6 Determination of short-chain fatty acids

Fecal short-chain fatty acids (SCFAs) were extracted and quantified using gas chromatography-mass spectrometry (GC-MS), following a previously described method with minor modifications (Ren et al., 2015). Briefly, 50 mg of fecal sample suspended in saturated sodium chloride (NaCl) solution. The suspension was acidified with 20 μL of sulfuric acid, and SCFAs were extracted using 1 mL of anhydrous ether. After centrifugation at 13,000×*g* for 15 min, the supernatant was transferred to a tube containing 0.25 g of anhydrous sodium sulfate to remove residual moisture. The upper organic layer was collected and analyzed by GC-MS.

### 2.7 Comparative genomic analysis and KEGG annotation

Genome sequencing of four *L. helveticus* strains (H1–H4) was performed using the Illumina NovaSeq platform. Reads were assembled into draft genomes using SPAdes (v3.15.4) (Bankevich et al., 2012), and open reading frames (ORFs) were annotated with Prokka (v1.14.6) (Seemann, 2014). Orthologous gene clusters were identified using OrthoFinder (v2.5.4) to distinguish core and strain-specific genes (Emms and Kelly, 2019). Unique genes from strain H3 were functionally annotated using the Kyoto Encyclopedia of Genes and Genomes (KEGG) database. KEGG Orthology (KO) assignments were performed using the KEGG Automatic Annotation Server (KAAS) (Moriya et al., 2007).

### 2.8 Statistical analysis

Statistical analyses were performed using the SPSS software (version 25.0). Significant differences among groups were assessed by one-way analysis of variance (ANOVA), followed by Tukey's post-hoc test for multiple comparisons. Data were presented as mean ± standard deviation (SD), and the statistical significance was defined as *P* < 0.05. Correlations between gut microbiota composition and SCFA concentrations were analyzed and visualized using HemI software (version 1.0) and GraphPad Prism (version 8.0.2).

## 3 Results

### 3.1 Alleviation of ETEC-induced diarrhea symptoms

**Fig. 2:**
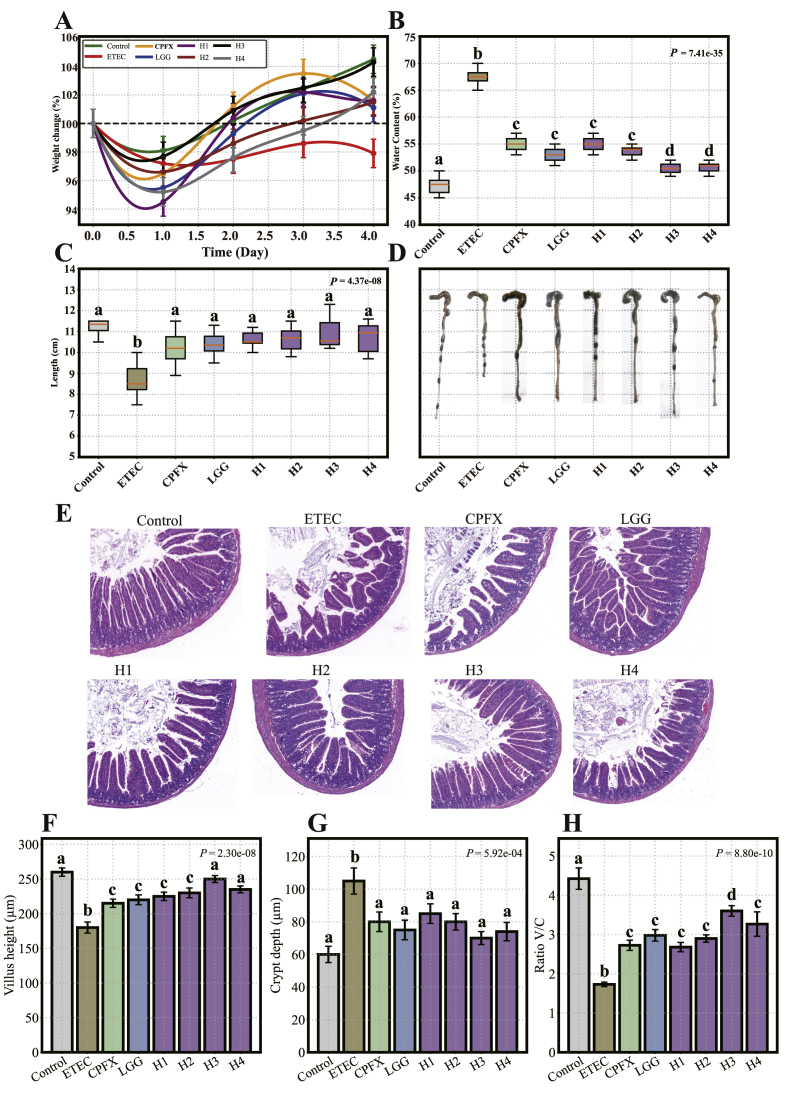
Impacts of L. *helveticus* on ETEC-induced diarrhea-related parameters. (A) Body weight variation, (B) Stool water content, (C) Colon length, and (D) Jejunal histological structure (scale bar = 200 μm), (F) Villus height, (G) Crypt depth, (H) Villus/Crypt. Different letters indicate significant differences between groups according to Tukey's post hoc test. Values are expressed as mean ± SD.

A mouse model of ETEC-induced diarrhea was employed to evaluate the effects of *L. helveticus* on body weight and fecal water content (Fig. 2A). The body weight of the control group 3.53 %, which was significantly higher than that of the ETEC group (*P* < 0.05). No significant differences in body weight were observed between the ETEC group and those treated with ciprofloxacin, LGG, *L. helveticus* strains (except for the strain H4) (*P* > 0.05).

Fecal water content in the ETEC group was significantly elevated compared to the control group (*P* < 0.001) (Fig. 2B). Treatment with ciprofloxacin, *L. rhamnosus* GG, and *L. helveticus* strains (H1–H4) significantly reduced fecal water content (*P* < 0.001), compared to the ETEC group. Among the L. *helveticus* strains, H3 demonstrated the most pronounced reduction in fecal water content.

In contrast, mice challenged with ETEC exhibited substantial damage to the jejunal morphology, including shortened and disorganized villi. Colon length was significantly reduced in the ETEC group (*P* < 0.05), compared to the control (Fig. S1). This reduction was significantly alleviated by the treatment with ciprofloxacin, *L. rhamnosus* GG, and *L. helveticus* strains (*P* < 0.05). Among these, the strain *L. helveticus* H3 exhibited the most notable recovery, with the colon length nearly restored to that of the control group (Fig. 2C and D). To assess the protective effects of *L. helveticus* against ETEC-induced jejunal injury, villus height, crypt depth, and the villus-to-crypt ratio were histologically evaluated (Fig. 2F–H). ETEC infection caused significant mucosal damage, with villus height reduced by 30.8 % and crypt depth increased by 75.0 % (both *P* < 0.001; Fig. 2F and G). Treatment with ciprofloxacin, LGG, and each of the *L. helveticus* strains (H1 to H4) significantly improved both parameters (*P* < 0.01). As a result, the villus-to-crypt ratio, which was markedly decreased by ETEC (*P* < 0.001), was significantly restored following intervention (*P* < 0.01; Fig. 2H). Among all treatments, strain H3 showed the greatest protective effect, with the most pronounced improvement in villus morphology.

### 3.2 Immunomodulatory effects of *L. helveticus* on ETEC-infected mice

#### 3.2.1 Suppression of pro-inflammatory cytokines by *L. helveticus*

**Fig. 3:**
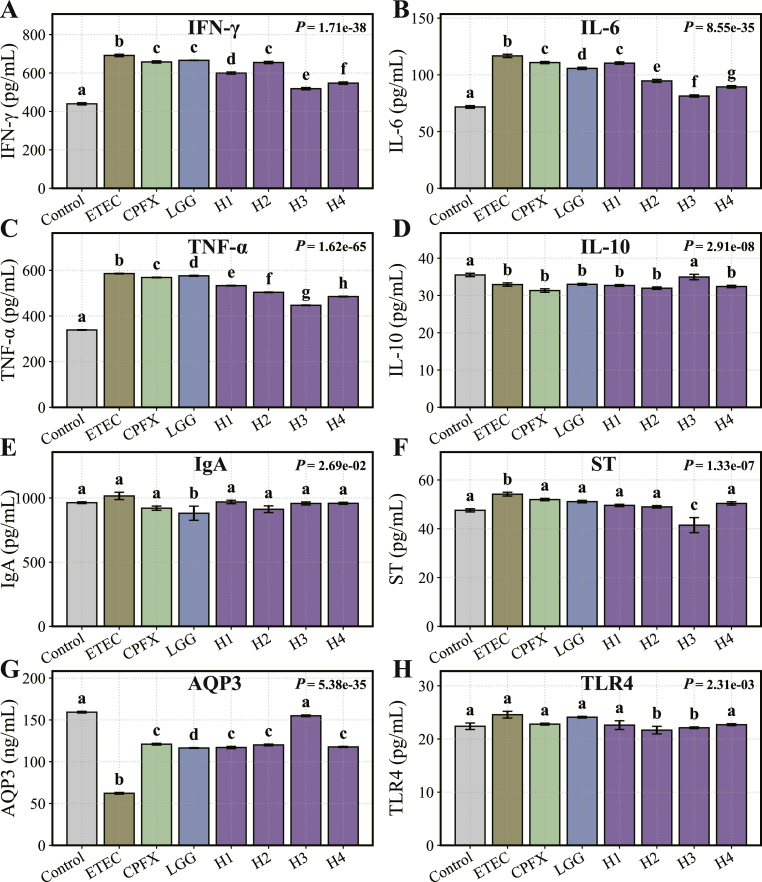
Modulatory effects of *L. helveticus* on inflammatory cytokines, ST, AQP3, and TLR4 expression levels. (A) Interferon-γ (IFN-γ), (B) Interleukin-6 (IL-6), (C) Tumor necrosis factor-α (TNF-α), (D) Interleukin-10 (IL-10), (E) Immunoglobulin A (IgA), (F) Heat-stable enterotoxin (ST), (G) Aquaporin 3 (AQP3) and (H) Toll-like receptor 4 (TLR4). Different letters indicate significant differences between groups according to Tukey's post hoc test. Values are expressed as mean ± SD.

To assess the effects of *L. helveticus* on immune cytokine regulation, serum levels of pro-inflammatory mediators and anti-inflammatory cytokines were measured in ETEC-challenged mice. ETEC exposure significantly elevated the levels of IL-6, TNF-α, and IFN-γ, with increases of at least 1.44-fold compared to the control group (*P* < 0.05, Fig. 3A–C). Treatment with ciprofloxacin reduced IL-6 and IFN-γ levels (*P* < 0.05), but had no significant effect on TNF-α. In contrast, all *L. helveticus* strains (H1–H4) significantly reduced IL-6, TNF-α, and IFN-γ levels compared to the ETEC group (*P* < 0.05), with effects similar to those of LGG treatment.

Moreover, ETEC exposure reduced the levels of the anti-inflammatory cytokine IL-10 (*P* < 0.05, Fig. 3D), while ciprofloxacin had no significant effect on IL-10. Notably, *L. helveticus* strain H3 significantly increased IL-10 levels (*P* < 0.05), whereas the other strains did not show significant changes. Serum IgA levels were not significantly altered by ETEC exposure (Fig. 3E), and none of the *L. helveticus* strains affected IgA levels. However, LGG treatment significantly decreased serum IgA (*P* < 0.05).

#### 3.2.2 Regulation of enterotoxin production and aquaporin expression by *L. helveticus*

ETEC exposure significantly increased serum levels of heat-stable enterotoxin (ST), which were elevated by 5.84 % compared to controls (*P* < 0.05, Fig. 3F). Ciprofloxacin did not affect ST levels compared to the ETEC group. Among the L. *helveticus* strains, H3 and H4 significantly reduced serum ST levels (*P* < 0.05), while the other strains and LGG had no significant effect.

To evaluate the influence of *L. helveticus* on intestinal water balance, colonic levels of aquaporin AQP3 were measured. ETEC exposure reduced colonic AQP3 levels by 60.89 % compared to the control group (*P* < 0.05, Fig. 3G). Neither ciprofloxacin nor LGG treatment significantly altered AQP3 levels in ETEC-challenged mice. However, strain H3 significantly increased colonic AQP3 levels (*P* < 0.05), while the other strains did not significantly affect AQP3 levels.

#### 3.2.3 Regulation of TLR4 expression by *L. helveticus* in ETEC-challenged mice

TLR4, a key receptor involved in immune responses and intestinal inflammation, was also evaluated as a marker of immune modulation. ETEC exposure resulted in a slight but non-significant increase in TLR4 expression (Fig. 3H). Ciprofloxacin and LGG treatments did not significantly affect TLR4 expression compared to the ETEC group. In contrast, strains H2 and H3 significantly decreased TLR4 expression (*P* < 0.05), while the other strains had no significant impact on TLR4 levels.

In summary, *L. helveticus* administration exerted significant immunomodulatory effects in ETEC-infected mice. All tested strains markedly reduced systemic levels of the pro-inflammatory cytokines IL-6, TNF-α, and IFN-γ, while strain H3 specifically increased the anti-inflammatory cytokine IL-10. Furthermore, strains H3 and H4 decreased serum levels of heat-stable enterotoxin, and H3 improved intestinal water balance by upregulating AQP3 expression. Notably, strains H2 and H3 downregulated TLR4 expression, suggesting strain-specific modulation of innate immune signaling.

### 3.3 *L. helveticus* regulates gut microbiota disrupted by ETEC infection

**Fig. 4:**
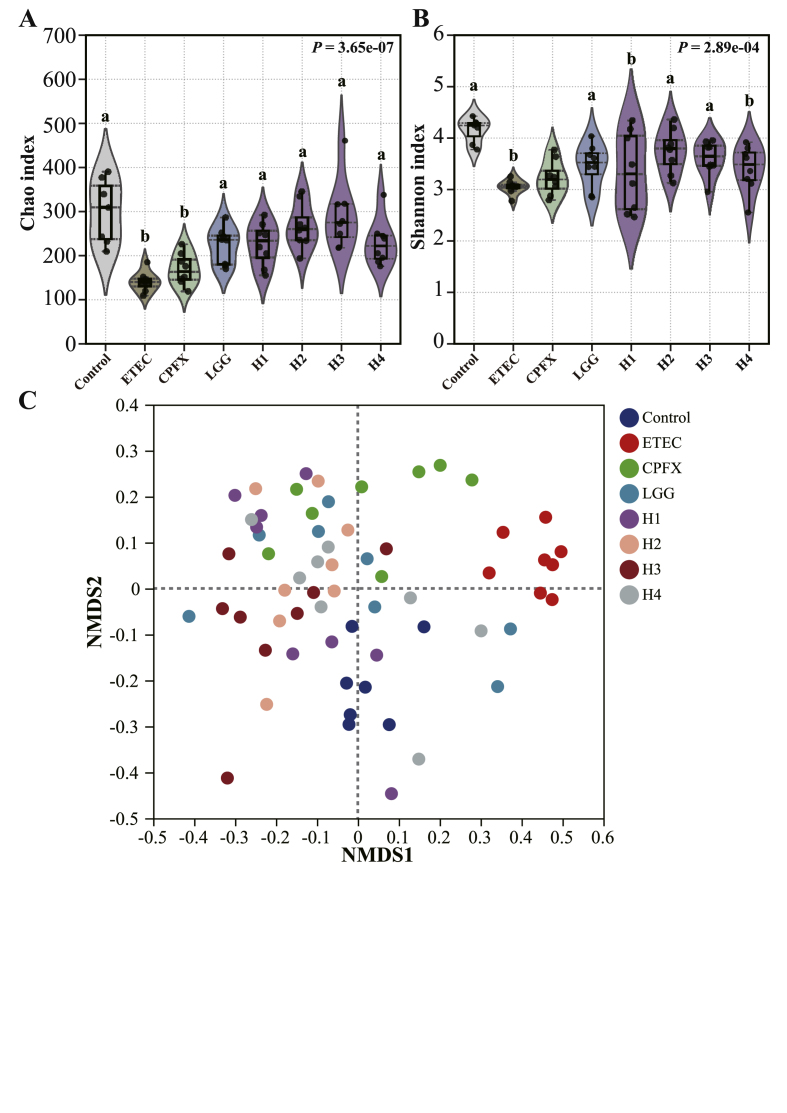
Regulatory effects of *L. helveticus* on gut microbial composition and diversity. (A) Chao1 richness index, (B) Shannon diversity index, and (C) NMDS analysis. Different letters indicate significant differences between groups according to Tukey's post hoc test. Values are expressed as mean ± SD.

To investigate the effects of *L. helveticus* on gut microbiota disturbances caused by ETEC infection, high-throughput sequencing was performed to analyze the gut microbiota composition. Compared to control mice, both Chao1 and Shannon indices significantly decreased in the ETEC group (*P* < 0.05, Fig. 4A and B). Ciprofloxacin and L. *rhamnosus* GG treatments both significantly elevated the Chao1 index (*P* < 0.05), but had no significant impact on the Shannon index. Treatment with all four *L. helveticus* strains significantly improved the Chao1 index compared to the ETEC group (*P* < 0.001), indicating increased species richness. However, only strains H2 and H3 significantly increased the Shannon index (*P* < 0.05). Non-metric multidimensional scaling (NMDS) analysis revealed distinct differences in microbiota profiles between ETEC-infected and control mice (Fig. 4C), with all four *L. helveticus* strains showing regulatory effects on microbial composition.

**Fig. 5:**
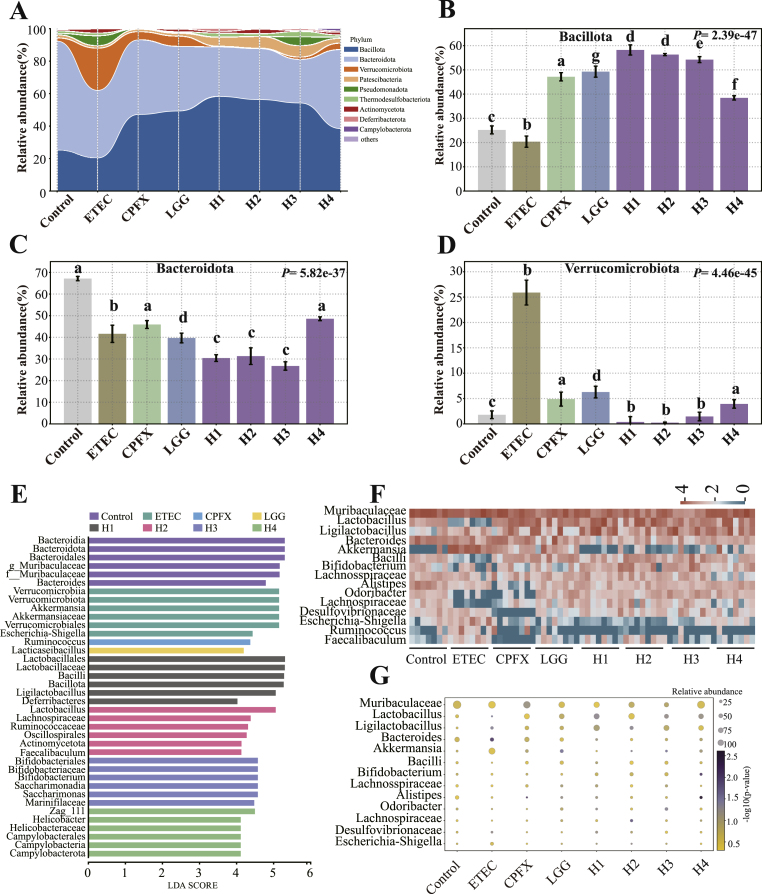
Effects of L. *helveticus* on gut microbiota. (A) Taxonomic distribution at the phylum level; (B) Comparison of the relative abundance of Bacillota; (C) Comparison of the relative abundance of Bacteroidota; (D) Comparison of the relative abundance of Verrucomicrobiota; (E) Histogram of taxa distribution based on LDA scores (log LDA score >3.0), with significantly enriched taxa displayed in the right panel; (F) Hierarchical clustering heatmap showing genus-level abundance in fecal samples; (G) Comparative analysis of dominant genera across groups. Different letters indicate significant differences between groups according to Tukey's post hoc test. Values are expressed as mean ± SD.

The gut microbiota of control mice was predominantly composed of Bacillota, Bacteroidota*,* and Verrucomicrobiota (Fig. 5A). ETEC infection significantly reduced the abundance of Bacillota and increased Verrucomicrobiota (*P* < 0.001, Fig. 5B–D). Treatment with ciprofloxacin, LGG, and *L. helveticus* H1–H3 significantly restored Bacillota levels (*P* < 0.05), while H4 had no significant effect. However, all treatments, including H4, significantly reduced Verrucomicrobiota abundance compared with the ETEC group (*P* < 0.05). No significant differences in Bacteroidota abundance were observed across groups (Fig. 5C).

Linear discriminant analysis effect size (LEfSe) identified genera with significant differences in relative abundance across groups (Fig. 5E). ETEC infection significantly reduced the abundance of *Lactobacillus*, Bacilli, and Odoribacter (*P* < 0.05), while substantially increasing the abundance of *Akkermansia* and *Escherichia-Shigella* (*P* < 0.05, Fig. 5F). Compared to the ETEC group, ciprofloxacin treatment increased the abundance of Ligilactobacillus and Lachnospiraceae, although these changes were not statistically significant (Fig. 5G). LGG treatment significantly elevated the abundance of Bacilli and *Lacticaseibacillus* (*P* < 0.05) while markedly reducing Odoribacter. Treatment with L. *helveticus* strains H1 and H2 significantly increased *Lactobacillus* levels. Notably, strain H3 significantly enhanced the abundance of *Bifidobacterium* and Bacilli, while reducing levels of Odoribacter, Muribaculaceae, *Akkermansia*, and *Escherichia-Shigella*.

### 3.4 Modulation of gut microbiota by *L. helveticus* promotes SCFA production and suppresses inflammation

**Fig. 6:**
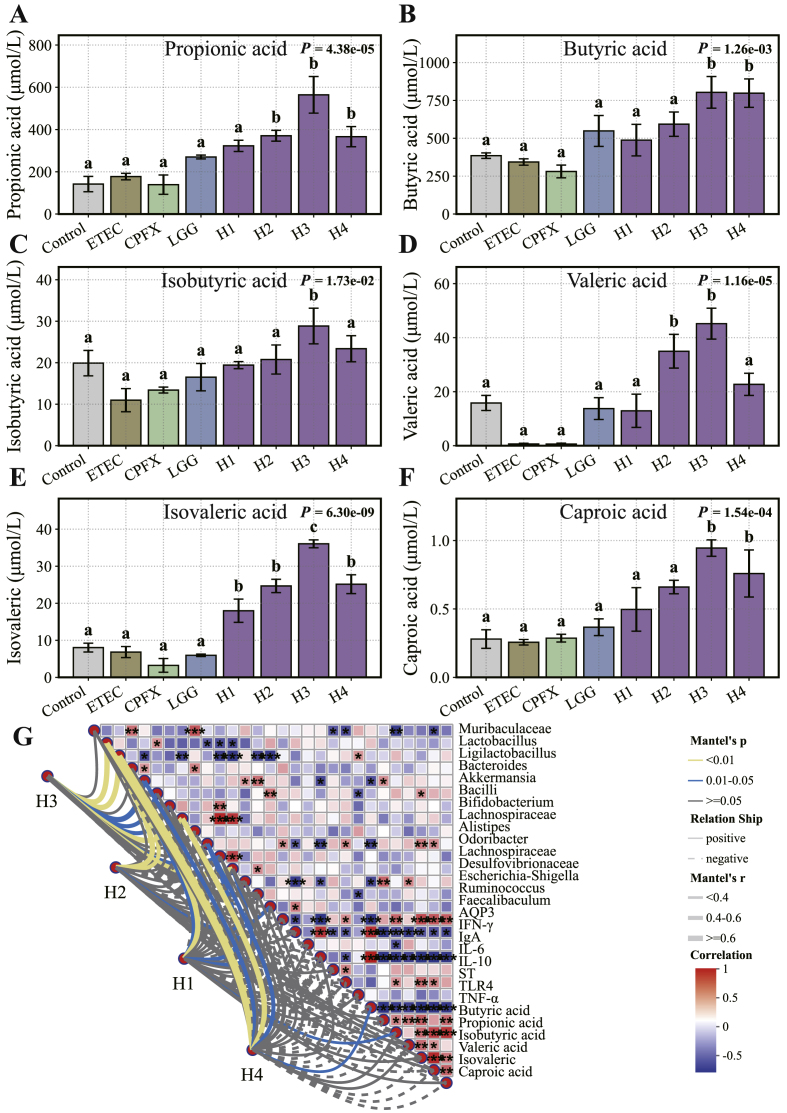
Effects of L. *helveticus* on key SCFAs. (A) Propionic acid, (B) Butyric acid, (C) Isobutyric acid, (D) Valeric acid, (E) Isovaleric acid, and (F) Caproic acid. (G) Mantel test-based correlation network and heatmap analysis linking gut microbiota, cytokines, and SCFAs in response to four *L. helveticus* strains (H1–H4). Different letters indicate significant differences between groups according to Tukey's post hoc test. Values are expressed as mean ± SD.

To investigate the effects of *L. helveticus* on short-chain fatty acid (SCFA) production, cecal contents were analyzed using gas chromatography-mass spectrometry (GC-MS) (Fig. 6A–F). Ciprofloxacin and LGG treatments did not significantly affect SCFA levels. In contrast, strain H3 significantly increased the concentrations of acetic, propionic, butyric, isobutyric, valeric, isovaleric, and caproic acids (*P* < 0.05). Strain H2 significantly elevated valeric and isovaleric acids (*P* < 0.05), while H4 increased butyric, isovaleric, and caproic acids. Strain H1 had no significant effect on SCFA levels.

Spearman's correlation analysis demonstrated that SCFA-associated genera *Lactobacillus*, *Odoribacter*, *Alistipes*, *Ligilactobacillus*, and *Bifidobacterium* were positively correlated with several SCFAs, including butyric, isobutyric, valeric, and isovaleric acids (*P* < 0.05, Fig. 6G). These genera also exhibited significant negative correlations with pro-inflammatory cytokines IFN-γ, IL-6, and TNF-α (*P* < 0.05), while *Lactobacillus* showed a positive correlation with AQP3. In contrast, *Akkermansia*, *Escherichia-Shigella*, and the *Ruminococcus gnavus* group were negatively correlated with most SCFAs and positively associated with pro-inflammatory cytokines (*P* < 0.05). Notably, *Escherichia-Shigella* showed strong positive correlations with IFN-γ, IL-6, and TNF-α.

### 3.5 Comparative genomic analysis identifies unique metabolic features in *L. helveticus* strain H3

**Fig. 7:**
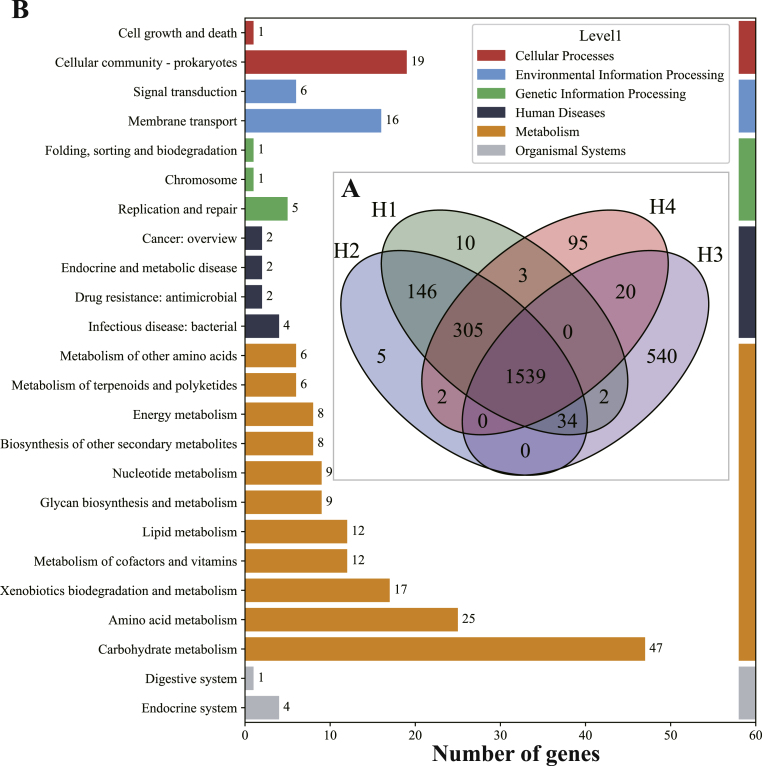
Comparative genomic analysis and KEGG functional annotation of four *L. helveticus* strains. (A) Venn diagram illustrating the core and strain-specific genes among H1, H2, H3, and H4. (B) KEGG pathway classification of H3-specific genes across major functional categories.

Comparative genomic analysis revealed that the four *L. helveticus* strains (H1–H4) shared 1539 core genes, while strain-specific genes varied significantly among them, with strain H3 exhibiting the highest number (540 unique genes) compared to strains H4 (95 genes), H1 (10 genes), and H2 (5 genes) (Fig. 7A). KEGG annotation of H3-specific genes demonstrated notable enrichment in metabolic pathways, especially carbohydrate metabolism (47 genes), amino acid metabolism (25 genes), and xenobiotics biodegradation (17 genes), as well as pathways related to environmental adaptation, such as membrane transport (16 genes) and cellular community (19 genes) (Fig. 7B). These results suggest that the unique metabolic and environmental adaptability genes in strain H3 may underpin its superior anti-diarrheal efficacy.

## 4 Discussion

ETEC remains a leading cause of diarrheal disease worldwide, particularly affecting children, travelers, and livestock (Ma et al., 2024; Prieto et al., 2022). The pathogenesis of ETEC is primarily driven by colonization factors and heat-stable enterotoxins, which disrupt intestinal epithelial function (Zhang et al., 2022). Although antibiotics such as ciprofloxacin are commonly used, concerns about resistance and side effects highlight the need for safer, more sustainable alternatives (Xue et al., 2009). This study identified *L. helveticus* as a promising probiotic capable of alleviating ETEC-induced diarrhea through multiple, strain-dependent mechanisms.

ETEC infection induced hallmark diarrheal features in mice, including weight loss, elevated fecal water content, villus atrophy, and colon shortening, confirming successful model establishment (Wang et al., 2016; Yu et al., 2016; Yang et al., 2021; Whon et al., 2021). Mechanistically, these symptoms were closely associated with disrupted intestinal fluid regulation and heightened inflammation. In particular, the downregulation of aquaporin 3 (AQP3), a membrane channel essential for water absorption, contributed to the elevated fecal moisture observed (Qadri et al., 2005; Zhu et al., 2017, 2023). In this study, *L. helveticus* H3 significantly restored AQP3 expression, indicating improved epithelial fluid transport and a reestablishment of intestinal water homeostasis.

Concurrently, ETEC challenge triggered robust upregulation of pro-inflammatory cytokines (IFN-γ, TNF-α, IL-6), consistent with NF-κB pathway activation (Dong et al., 2020; Tsai et al., 2012; Huang et al., 2012). These cytokines impair epithelial integrity and amplify tissue injury. Treatment with L. *helveticus* significantly suppressed these inflammatory mediators, indicating strong immunomodulatory activity. Although mucosal IgA levels remained unchanged, this result is consistent with previous reports indicating that probiotic effects on IgA production are both strain-specific and context-dependent (Bae et al., 2003; Ten Bruggencate et al., 2015). Meanwhile, *L. helveticus* H1 and H2 significantly downregulated TLR4 expression, further suppressing NF-κB signaling. Together, these findings indicate that *L. helveticus* exerts multifaceted protection during ETEC-induced intestinal stress by restoring fluid balance, preserving epithelial barrier integrity, and attenuating inflammation through TLR4–NF-κB axis modulation.

Villus height and crypt depth are key structural indicators of intestinal health and play critical roles in maintaining mucosal homeostasis (Goto et al., 2022). In this study, *L. helveticus* treatment preserved jejunal architecture by increasing villus height and reducing crypt depth following ETEC challenge. These results align with previous reports showing that *Lactobacillus* strains can improve intestinal morphology by promoting villus elongation and mitigating crypt hyperplasia (Chen et al., 2023; Zhang et al., 2025), suggesting the potential of *L. helveticus* as a protective probiotic against enteric injury.

Ciprofloxacin, while effective in reducing ETEC burden as a broad-spectrum antibiotic, exerts non-selective bactericidal effects that may disrupt commensal microbiota and delay mucosal recovery (Zimmermann and Curtis, 2019). This disruption could explain the only partial improvements observed in body weight, ileal architecture, and inflammatory cytokine expression. Similarly, although *L. rhamnosus* GG is a widely studied probiotic with recognized immunomodulatory and barrier-supporting properties, its efficacy appears to be context- and strain-dependent (Steele, 2022). In this acute infection model, LGG did not fully restore mucosal integrity or suppress proinflammatory signaling, possibly due to limited colonization or insufficient modulation of the dysbiotic gut environment. These findings highlight the complex interplay between host, microbiota, and pathogen during enteric infection (Bäumler and Sperandio, 2016) and suggest that *L. helveticus* H3, which demonstrated superior mucosal protection and immune modulation, may offer distinct therapeutic advantages. Compared to ciprofloxacin and LGG, H3 consistently outperformed in alleviating intestinal injury, supporting its potential as a targeted intervention in acute diarrheal disease.

Gut microbial dysbiosis is a known consequence of ETEC infection (Sun et al., 2019). NMDS analysis revealed significant changes in microbial composition post-infection, including an increase in *Akkermansia* and *Escherichia–Shigella* and a reduction in *Lactobacillus* and *Bifidobacterium*. Treatment with L. *helveticus* restored microbial balance by reducing pathogens and enriching beneficial taxa. Although *A*. *muciniphila* is widely recognized as a beneficial commensal due to its roles in metabolic regulation, mucosal protection, and immune modulation (Liu et al., 2022). In this study, *A. muciniphila* was significantly enriched in the ETEC-infected group, coinciding with aggravated intestinal injury and disruption of the mucus barrier. This observation is consistent with emerging evidence that *A. muciniphila* overgrowth during enteric infection may aggravate disease by eroding the mucus barrier and triggering pathogen virulence, thereby intensifying mucosal inflammation (Wang et al., 2025). Alternatively, its expansion may reflect a host-driven compensatory response aimed at restoring mucus homeostasis, given its capacity to promote mucin renewal and modulate immune tone in non-infectious settings (Steimle et al., 2024). Collectively, these findings underscore the functional plasticity of *A. muciniphila* and challenge its characterization as universally beneficial. They also emphasize the importance of evaluating probiotic function within specific disease contexts, as microbial roles may shift from symbiotic to pathogenic depending on host physiology and environmental conditions. Further mechanistic investigations are needed to elucidate the factors that regulate this transition.

A key feature of probiotic efficacy is the modulation of microbial metabolites. Short-chain fatty acids (SCFAs), particularly butyrate, are essential for maintaining intestinal barrier function and inhibiting pathogen colonization (Asahara et al., 2001; Eeckhaut et al., 2011; Pasam et al., 2025). *L. helveticus* H3 significantly elevated SCFA levels, particularly acetic, propionic, and butyric acids. Correlation analysis linked these increases to higher abundances of SCFA-producing genera, such as *Odoribacter*, *Alistipes*, *Ligilactobacillus*, and *Bifidobacterium*. These genera were inversely correlated with pro-inflammatory cytokines, reinforcing the idea that microbial metabolic activity underpins the anti-inflammatory effects of probiotics. Importantly, strain specificity emerged as a critical factor. While H3 showed the most potent effects on SCFA enhancement and cytokine suppression, other strains demonstrated differential regulatory profiles. This may be attributed to strain-specific differences in exopolysaccharide production, surface protein expression, and metabolite secretion among *L. helveticus*, which in turn result in variations in functional performance (Wang et al., 2022).

Genomic divergence among probiotic strains can significantly influence their functional attributes (Chang et al., 2024). In this study, *L. helveticus* H3 exhibited the largest genome size, substantially exceeding H1, H2, and H4 (Table 1). This genomic expansion includes 540 unique genes, considerably more than those in other strains, indicating H3's enhanced adaptive capacity within the intestinal environment and its potential role in diarrhea mitigation. The enrichment of H3-specific genes in carbohydrate and amino acid metabolism pathways may facilitate the production of metabolites such as butyrate and GABA, which support gut barrier integrity and mitigate inflammation through NF-κB signaling modulation (Deng et al., 2023; Lu et al., 2022). Additionally, the presence of 17 xenobiotic biodegradation genes suggests an increased capacity for toxin metabolism, potentially reducing ETEC-induced gut damage. Moreover, 16 membrane transport and 19 cellular community genes indicate enhanced nutrient acquisition, waste elimination, and competitive colonization, promoting H3's persistence in the gut. Genes involved in DNA repair, signal transduction, and stress response may further strengthen its resilience against gastrointestinal stressors, maintaining probiotic viability. Collectively, the genomic expansion in H3, characterized by metabolic versatility, detoxification capability, and environmental adaptation, likely contributes to its superior anti-diarrheal efficacy, positioning it as a promising probiotic candidate for ETEC-related diarrhea intervention. These findings highlight the therapeutic potential of *L. helveticus* as a multifaceted probiotic for diarrheal diseases, with implications for clinical and agricultural applications. Further studies are warranted to assess its functional capacity and long-term efficacy comprehensively.

## 5 Conclusion

This study establishes *L. helveticus* as a potent probiotic for mitigating ETEC-induced diarrhea, with distinct strain-specific mechanisms underlying its protective effects. Treatment with L. *helveticus* strains H1–H4 effectively alleviated pathological features such as weight loss, intestinal damage, immune dysregulation, and SCFA reduction, likely through multiple pathways, including enterotoxin suppression, aquaporin-mediated water balance restoration, SCFA enhancement, and attenuation of inflammatory signaling. Notably, genomic analysis revealed that strain H3 harbors 540 unique genes involved in carbohydrate and amino acid metabolism, xenobiotic degradation, and membrane transport, potentially contributing to its superior SCFA production, toxin detoxification, and colonization capacity. These genetic attributes, coupled with H3's pronounced modulation of cytokine expression and microbiota composition, position it as a promising probiotic candidate for managing infectious diarrhea. Further investigations are warranted to elucidate its functional mechanisms and assess its clinical and agricultural applications.

## CRediT authorship contribution statement

**Zhen Zhang:** Investigation, Data curation, Formal analysis, Writing – original draft. **Jianmin Lv:** Investigation, Writing – review & editing. **Xin Wang:** Visualization, Validation, Formal analysis, Supervision. **Ling Chun:** Investigation, Methodology. **Qiannan Yang:** Data curation, Formal analysis. **Huarui Zhao:** Writing – review & editing. **Siming Xue:** Writing – review & editing. **Ziyi Zhang:** Writing – review & editing. **Xiaobo Liu:** Conceptualization, Supervision. **Shiwei Wang:** Conceptualization, Funding acquisition, Supervision. **Yanmei Sun:** Conceptualization, Resources, Project administration.

## Declaration of competing interest

The authors declare the following financial interests/personal relationships which may be considered as potential competing interests: Yanmei Sun reports financial support was provided by Shaanxi Fundamental Science Research Project for Chemistry & Biology. Yanmei Sun reports financial support was provided by 10.13039/501100001809National Natural Science Foundation of China. Xiaobo Liu serves as Editor and Board Member of Current Research in Food Science, and had no involvement in the peer review of this article. If there are other authors, they declare that they have no known competing financial interests or personal relationships that could have appeared to influence the work reported in this paper.

## Data Availability

Data will be made available on request.
