# Supplementary material for: Lactobacillus helveticus mitigates diarrhea and inflammation induced by enterotoxigenic E. coli through rebalance of gut microbiota
Source: Curr Res Food Sci. 2025 Jul 17;11:101147. doi: 10.1016/j.crfs.2025.101147 (PMC12305323; doi:10.1016/j.crfs.2025.101147)
Supplement: Multimedia component 1 [file mmc1.docx]

*Lactobacillus helveticus* mitigates diarrhea and inflammation induced by enterotoxigenic *E. coli* through rebalance of gut microbiota

Zhen Zhang ^1,^ ^#^, Jianmin Lv ^2,^ ^#^, Xin Wang ^1^, Ling Chun ^1^, Qiannan Yang ^1^, Huarui Zhao ^1^, Siming Xue ^1^, Ziyi Zhang ^1^, Xiaobo Liu ^3,^ ^*^, Shiwei Wang ^1,^ ^*^, Yanmei Sun ^1,^ ^*^

^1^ Key Laboratory of Resource Biology and Biotechnology in Western China, Ministry of Education, Provincial Key Laboratory of Biotechnology, College of Life Sciences, Northwest University, 229 Taibai North Road, Xi’an, Shaanxi 710069, China.

^2^ Rehabilitation Science Institute, Shaanxi Provincial Rehabilitation Hospital, Xi'an, Shaanxi, 710065, China

^3^ Key Laboratory of Metabolic Engineering and Biosynthesis Technology, Ministry of Industry and Information Technology, Nanjing University of Science and Technology, Nanjing, Jiangsu 210094, China

**^#^** These authors contributed equally to this work.

* Corresponding author:

**Xiaobo Liu:** [xbliu@njust.edu.cn](mailto:xbliu@njust.edu.cn)

**Shiwei Wang:** [wangsw@nwu.edu.cn](mailto:wangsw@nwu.edu.cn)

**Yanmei Sun:** sun[yanmei@nwu.edu.cn](mailto:yanmei@nwu.edu.cn)

**
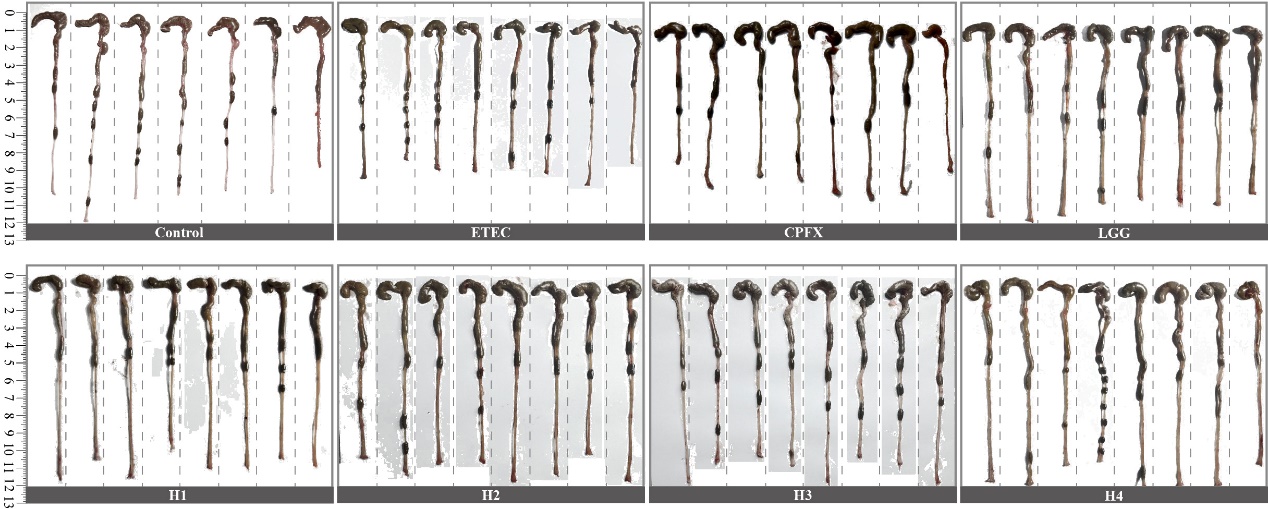
**

**Figure S1. Colon length images for each treatment group.** Colons (n = 8 per group) were excised, straightened beside a centimeter ruler (0–13 cm scale at left), and photographed. Dashed vertical lines separate individual specimens within Control, ETEC, CPFX, LGG, H1, H2, H3 and H4 group.
